# Procedural times in early non-intubated VATS program - a propensity score analysis

**DOI:** 10.1186/s12871-021-01270-4

**Published:** 2021-02-11

**Authors:** Isabella Metelmann, Johannes Broschewitz, Uta-Carolin Pietsch, Gerald Huschak, Uwe Eichfeld, Sven Bercker, Sebastian Kraemer

**Affiliations:** 1 Department of Visceral, Transplant, Thoracic and Vascular Surgery, University Hospital of Leipzig, Liebigstrasse 20, 04103 Leipzig, Germany; 2 Department of General, Visceral, Thoracic and Vascular Surgery, Faculty of Health Sciences Brandenburg, Brandenburg Medical School, University Hospital Neuruppin, Fehrbelliner Strasse 38, 16816 Neuruppin, Germany; 3 Department of Anesthesiology and Intensive Care Medicine, University Hospital of Leipzig, Liebigstrasse 20, 04103 Leipzig, Germany; 4 OR Management, University Hospital of Leipzig, Liebigstrasse 20, 04103 Leipzig, Germany

**Keywords:** VATS, Non-intubated VATS, Spontaneous ventilation, Video-assisted thoracoscopic surgery, Procedural times

## Abstract

**Background:**

Non-intubated video-assisted thoracic surgery (NiVATS) has been introduced to surgical medicine in order to reduce the invasiveness of anesthetic procedures and avoid adverse effects of intubation and one-lung ventilation (OLV). The aim of this study is to determine the time effectiveness of a NiVATS program compared to conventional OLV.

**Methods:**

This retrospective analysis included all patients in Leipzig University Hospital that needed minor VATS surgery between November 2016 and October 2019 constituting a NiVATS (*n* = 67) and an OLV (*n* = 36) group. Perioperative data was matched via propensity score analysis, identifying two comparable groups with 23 patients. Matched pairs were compared via t-Test.

**Results:**

Patients in NiVATS and OLV group show no significant differences other than the type of surgical procedure performed. Wedge resection was performed significantly more often under NiVATS conditions than with OLV (*p* = 0,043). Recovery time was significantly reduced by 7 min (*p* = 0,000) in the NiVATS group. There was no significant difference in the time for induction of anesthesia, duration of surgical procedure or overall procedural time.

**Conclusions:**

Recovery time was significantly shorter in NiVATS, but this effect disappeared when extrapolated to total procedural time. Even during the implementation phase of NiVATS programs, no extension of procedural times occurs.

## Background

Non-intubated video-assisted thoracic surgery (NiVATS) has been introduced to surgical medicine in order to reduce the invasiveness of anesthetic procedures. NiVATS has the potential to reduce operating time and length of hospital stay by a faster recovery after thoracic surgery [1, 2]. These advances seem to derive from avoiding adverse effects of intubation and one-lung ventilation (OLV). OLV is known to increase the risk of lung injury due to high tidal volumes causing high shear stress and strain, loss of functional residual capacity, oxidative stress, overhydration as well as re-expansion injury [1]. It has been shown that even subclinical lung injury can cause postoperative complications [2]. Furthermore, in contrast to NiVATS, OLV requires deep anesthesia with suppression of spontaneous breathing and muscle relaxation, thus posing an immanent risk of drug overdosing [3].. The absence of relaxation has the potential to reduce respiratory complications [2] while surgery during spontaneous breathing has the potential to worsen surgical conditions. The insertion of a double-lung tube (DLT) also increases the risk for oral, mucosal or dental injuries as well as postoperative sore throat [4].

Anesthesiologic management differs substantially regarding the degree of sedation associated with the surgical procedure performed. Patterns indicate that mainly minor VATS like wedge or peripheral nodule resections are performed in awake or minimally sedated patients, while segmentectomy or lobectomy mostly ask for deeper sedation [2]. To facilitate different operations a variety of analgesic concepts has been described including thoracic epidural anesthesia, paravertebral block, and intercostal block.

All these procedures ask for a well-coordinated protocol concerning criteria for indication and contraindication and the appropriate anesthesiologic handling including criteria for conversion to general anesthesia. Hence, interdisciplinary communication is crucial and implementation processes can be demanding in procedural time and use of resources. Surgical and anesthesiologic expertise with VATS procedures, as well as precise interdisciplinary communication, are major preconditions for the successful implementation of NiVATS. Thus, minor VATS procedures such as wedge resections, pleurectomy, sympathectomy, pleurodesis with talcum or evacuation of hemothorax serve as good starting points for NiVATS programs.

While the pathophysiologic benefits from spontaneous ventilation in general seem conclusive, the evidence level of the advantages of NiVATS remains quite low [5]. Most studies on NiVATS focus on safety and clinical outcomes in comparison to OLV. The aim of this study is to determine the time effectiveness of a NiVATS program compared to conventional OLV.

## Material and methods

### Study design and statistical analysis

Ethical approval for this retrospective evaluation of archived, pseudonymized patient data was granted from the Scientific Ethical Committee at the Medical Faculty, Leipzig University (ref. no. 399/19). The study was conducted in compliance with the International Conference on Harmonization Guidelines for Good Clinical Practice and the principles of the Declaration of Helsinki.

Patient data was retrieved from the documentation system of Leipzig University Hospital. All patients that received minor VATS surgery between November 2016 and October 2019, performed as either OLV (*n* = 36) or NiVATS (*n* = 67) procedure, were considered for this investigation. To reduce selection bias between the two groups perioperative data was matched via propensity score analysis. Based on that, two comparable groups with 23 patients each were identified. Matched pairs were compared via t-Test. Analysis was performed using SPSS Version 24 (IBM).

Time effectiveness was measured by duration of surgery, time for induction of anesthesia, recovery time and overall procedural time. Duration of surgery is defined as time from incision to suture. Time for induction of anesthesia means the period from the first injection or penetration of the skin until the patient is ready for surgical preparation. Recovery time is defined by the time from suture to extubation or relief from laryngeal mask. Overall procedural time means the sum of the three aforementioned periods.

### Eligibility criteria for VATS procedure

All patients selected for VATS procedure met the following inclusion criteria: American Society of Anesthesiologists risk classification (ASA) I-III, age older than 18 years and body mass index less or equal 30 kg/m^2^. Exclusion criteria for NiVATS procedure were defined as New York Heart Association (NYHA) stages III or IV, increased risk for aspiration, pacemaker, pregnancy and lactation period, neuromuscular diseases, and contraindication for regional anesthesia.

### Anesthesia

Patients in both groups underwent general anesthesia. Patients in the NiVATS group were treated under spontaneous ventilation with laryngeal mask, while patients in the OLV group received surgery with double-lumen endotracheal intubation.

In NiVATS group, after induction with propofol and remifentanil anesthesiologic management included a balanced anesthesia with sevoflurane/remifentanil, ventilation via laryngeal mask and regional anesthesia with erector spinae plane block or intercostal blockade where appropriate (*n* = 23). Ultrasound-assisted application of regional anesthesia took 10 min time on average. Regional anesthesia for NIVATS was aiming at facilitating spontaneous breathing by reducing opioid doses. Twelve patients received a patient-controlled analgesia (PCA) pump with piritramide for postoperative analgesia. OLV group management included balanced anesthesia with sevoflurane/sufentanil and induction with propofol, sufentanil and rocuronium. Additional regional anesthesia was rare in OLV group with only 4 patients receiving a peridural catheter with ropivacaine/sufentanil. In some cases, PCA pump with piritramide was implemented for postoperative analgesia (*N* = 12). DLT was inserted under videolaryngeoscopic view. Routine monitoring consisted of ECG, pulse oximetry and invasive blood pressure and relaxometry. The fiberscopic or endoscopic control of the tube position was performed after lateral positioning. Lateral position was the same in both groups. Patients were extubated right after the surgical procedure and transferred to post-anesthesia care unit.

## Results

### Patient characteristics of NiVATS and OLV group

Characteristics of the matched pair groups are shown in Table 1. As a result of matching patients in NiVATS and OLV group showed no significant differences other than the type of surgical procedure performed. Wedge resection was performed significantly more often under NiVATS conditions than with OLV (*p* = 0,043).

**Table 1:** Characteristics of groups after matching

Variable	NiVATS group (***n*** = 23)	OLV group (***n*** = 23)	***P*** value
**Age (in years)**	55,43 ± 18,713	57,83 ± 18,12	0,662
**Gender (M/F)**	13/10	14/9	0,765
**Body mass index (BMI) (in kg/m**^**2**^**)**	25,13 ± 4565	26,37 ± 4,38	0,35
**ASA physical status class (N [%])**			0,501
I	3 (13,04)	1 (4,35)	
II	11 (47,82)	14 (60,87)	
III	8 (34,78)	8 (34,78)	
IV	1 (4,35)	0	
**Smoking status (N [%])**			0,945
Smoker	6 (26,09)	7 (30,43)	
Non-Smoker	14 (60,82)	13 (56,52)	
Ex-Smoker	3 (13,04)	3 (13,04)	
**Smoking pack years**	9,7 ± 18,852	10,4 ± 16,225	0,887
**Comorbidity (N [%])**			
Arterial hypertension	9 (39,13)	10 (43,48)	0,765
Coronary Heart Disease	1 (4,35)	2 (8,69)	0,55
COPD	2 (8,96)	2 (8,96)	1,0
Diabetes mellitus	4 (17,39)	4 (17,39)	1,0
**Surgical location (Left/Right Lung/both (N [%])**	10 (43,48) / 12 (52,17) /1 (4,35)	10 (43,48)/13 (56,52)/0	0,595
**Reason for surgery**			0,059
Suspect nodule	12 (52,17)	20 (86,95)	
Pneumothorax	6 (26,09)	2 (8,96)	
Hematothorax	1 (4,35)	0	
Hyperhidrosis	1 (4,35)	0	
Interstitial lung disease	2 (8,96)	1 (4,35)	
Malign effusion	1 (4,35)	0	
**Type of surgical procedure**			0,042*
Wedge resection	14 (60,82)	21 (91,3)	
Pleurectomy and wedge resection	7 (30,43)	2 (8,96)	
Evacuation of the hematoma	1 (4,35)	0	
Sympathectomy	1 (4,35)	0	
**Length of Hospital stay (in days)**	3,9 ± 1,64	4,1 ± 1,13	0,594

No mortality occurred in either of the groups. No conversions of anesthetic or surgical procedures were needed. There were no cases of intraoperative aspiration, postoperative pulmonary edema, or pneumonia. All patients were monitored in the recovery room postoperatively. Mean duration of chest tube was 3 days postoperatively.

### Procedural times of NiVATS and OLV group

Table 2 shows the comparison of procedural times in NiVATS and OLV VATS after matching. Time between suture and end of anesthesia (recovery time) is significantly reduced by 7 min (*p* = 0,000) in the NiVATS group. There is no significant difference in the time for induction of anesthesia, duration of surgical procedure or overall procedural time.

**Table 2:** Procedural times in minutes of VATS in comparison after matching

Variable	NiVATS group (***n*** = 23)	OLV group (***n*** = 23)	Difference in minutes	***P*** value
**Duration of surgery**	49,96 ± 23,149	51,33 ± 17,423	− 1391	0,819
**Time for induction of anesthesia**	22,78 ± 12,124	21,39 ± 8,68	1391	0,657
**Recovery time**	10,04 ± 5858	17,09 ± 6222	− 7043	0,000**
**Overall procedural time**	83,22 ± 30,133	89,83 ± 21,582	− 6609	0,398

## Discussion

Our findings show that recovery time is significantly reduced when VATS is performed under NiVATS conditions, maybe due to deeper anesthesia for OLV. However, NiVATS does not lead to a reduction of preparation time or duration of surgery. Total procedural time of NiVATS and OLV therefore does not differ significantly.

We expected the more complex placement of DLT with potentially bronchoscopic position control and more extensive monitoring devices for general anesthesia to result in an extended preparation time of OLV compared to anesthesia in NiVATS settings.

However, in this patient cohort, additional regional anesthesia was performed more often than during OLV (*n* = 4), which may explain a more time-consuming preparation in NiVATS group than expected. Comparability of the procedural times may be weakened by that.

To our knowledge, there are only few studies on procedural times in NiVATS, all of them showing equal or even shorter anesthesia and overall procedural time in comparison to OLV [6–9]. Lan et al. [9] and Liu et al. [10] have found that NiVATS leads to faster postoperative re-convalescence and shorter hospital stay in a propensity score matched trial. Our findings seem contradictory to the findings of Lan et al. that described shorter operative and anesthesia duration in NiVATS. This difference may be explained by the inhomogeneity in surgical procedures and teams in our trial, while Lan et al. investigated lobectomy only [9].

Surgery during spontaneous breathing can be challenging, not only because of a non-collapsing lung but as well from strong excursions of the diaphragm. From our experience, disruptive influence from these conditions is lowest in resection of apical and superficial nodules. Hence, this might an important selection criterion from the surgeon’s point of view. Regardless potential concerns on increased complications due to the use of laryngeal masks, we have seen no related intra- or postoperative complications. In particular, no cases of aspiration, pneumonia or pulmonary edema occurred. A reason for that may be that all included operations were elective surgeries performed in fasted patients meaning no increased risk of aspiration [11]. Additionally, small extent of surgery and sufficient postoperative analgesia enabled patients for early mobilization reducing the risk for postoperative pneumonia.

Propensity score matching allows to counterbalance selection bias in non-randomized trials [12]. By that, we were able to offset our model for patient’s characteristics that commonly interfere with postoperative outcome, like age, ASA status, BMI, and smoking pack years. However, our groups differ significantly concerning the type of surgical procedure which may limit the explanatory power of our study. However, since all the procedures are similar concerning the surgical extent and duration, we assume this discrepancy to be negligible.

The following limitations must be stated concerning our trial: First, as we conducted a single-center retrospective study results may be difficult to transfer to other settings and resulted in inconsistent base line parameters, e.g., the use of different opioids. Second, results may be affected by the simultaneously introduced analgesic technique of erector spinae block, that may have led to an extension of preparation time probably compensating the time saved from placement of laryngeal mask. Third, due to the high staff turnover in university settings, we were not able to match data concerning surgical but particularly anesthesia teams. Changes in staff might have had a considerable impact on procedural times. Fourth, while propensity score matching serves to lessen selection bias, this is only applicable for already known and presumed confounding founders [12]. Unknown confounders that may interfere with the comparability of NiVATS and OLV can only be examined in randomized controlled trials.

## Conclusions

Comparison of procedural times in matched pairs showed a reduction of recovery time in NiVATS group. This effect disappeared when extrapolated to total procedural time. Our findings show that even during the implementation phase of NiVATS programs no extension of procedural times occurs.

## Data Availability

The datasets used and analyzed during the current study are available from the corresponding author on reasonable request.

## Abbreviations

ASA: American Society of Anesthesiologists risk classification
BMI: Body mass index
DLT: Double-lung tube
NiVATS: Non-intubated video-assisted thoracoscopic surgery
OLV: One-lung ventilation
PCA: Patient-controlled analgesia
